# Editorial: From adolescence to adulthood: the role of diet in preventing metabolic and mental health disorders

**DOI:** 10.3389/fnut.2026.1771520

**Published:** 2026-02-09

**Authors:** Desirée Victoria-Montesinos, José Francisco López-Gil, Ana María García-Muñoz

**Affiliations:** 1 Faculty of Pharmacy and Nutrition, UCAM Universidad Católica de Murcia, Murcia, Spain; 2 School of Medicine, Universidad Espíritu Santo, Samborondón, Ecuador; 3 Vicerrectoría de Investigación y Postgrado, Universidad de Los Lagos, Osorno, Chile

**Keywords:** adolescents, diet quality, Mediterranean diet, mental health, metabolic health

Adolescence is a pivotal developmental window during which dietary habits, psychosocial trajectories, and biological maturation converge to shape lifelong health. These years are marked by increasing autonomy over food choices alongside profound metabolic, neuroendocrine, and emotional changes, positioning adolescence as both a period of vulnerability and a powerful opportunity for prevention. In launching the Research Topic “*From adolescence to adulthood: the role of diet in the prevention of metabolic and mental health disorders*,” our aim was to integrate multidisciplinary evidence on how adolescent diet influences metabolic and psychological outcomes later in life.

This Research Topic brings together 10 contributions, including original research, cross-sectional epidemiological studies, and a scoping review, spanning diverse geographical and socioeconomic contexts, from Pakistan and Poland to Peru and Malaysia. Despite their heterogeneity, all contributions converge on a shared premise: adolescent diet is a modifiable determinant of obesity, cardiometabolic disease, mental health disorders, and disordered eating as individuals transition into early adulthood.

Collectively, the studies by Falak et al., Cuyan-Zumaeta et al., and Di Nucci et al. illustrate how dietary quality, emotional wellbeing, and broader lifestyle behaviors interact to shape adolescent health trajectories.

Several studies underscore the strong influence of socioeconomic and psychosocial factors on adolescent dietary quality. Evidence from South Asia shows that poor diet quality clusters among girls, younger adolescents, and those from lower socioeconomic backgrounds, and is closely linked to poorer academic performance and body image dissatisfaction, as reported by Falak et al. Comparable patterns are observed in European adolescents in the work by Żwirska et al., where snacking behaviors, fast food consumption, and dietary restraint vary markedly by BMI category. Importantly, higher fast food consumption among underweight adolescents challenges the assumption that lower body weight reflects healthier dietary behaviors, highlighting the complexity of adolescent nutrition.

These gradients extend beyond Europe. In a study by Shahridzal et al. among Malaysianadolescents with overweight and obesity reveals critically low adherence to fruit and vegetable intake recommendations, shaped by personal motivation, parental practices, and food availability within the home. Together, these findings demonstrate that adolescent dietary behavior cannot be separated from social inequality, family environment, and perceived body image.

Dietary behavior during adolescence is also closely intertwined with mental health. Collectively, the contributions support a bidirectional model in which diet quality, emotional wellbeing, and weight status reinforce one another. Adolescents consuming higher-quality carbohydrates exhibit lower levels of depression and anxiety, as shown by Ülger et al., while meal skipping is associated with higher anxiety symptoms. Emotional eating further emerges as a key behavioral pathway linking psychological distress to higher BMI, with emotional eating and mental wellbeing exerting opposing yet similarly sized influences on weight status, as reported by Cuyan-Zumaeta et al..

Across these dimensions, parents consistently emerge as key mediators of adolescent dietary trajectories. Large multinational data from Castellano et al. show that higher parental food literacy, fewer perceived barriers, stronger healthy-eating attitudes, and greater perceived enablers are associated with better adherence to protective dietary patterns, such as the Mediterranean diet, among children and adolescents. Framed within the COM-B model, these findings highlight parental empowerment as a scalable strategy for improving adolescent diet quality.

More specialized contributions further enrich this perspective. Research on parental knowledge, attitudes, and practices regarding metabolically healthy obesity in the work by Zhong et al. indicates that socioeconomic status, particularly income, strongly predicts parental engagement and understanding. At the population level, long-term analyses by Tian et al. document steady increases in the incidence and prevalence of anorexia nervosa among adolescents and young adults, particularly among females and younger cohorts, reflecting broader sociocultural pressures. Although not diet-specific, these trends unfold within increasingly obesogenic and diet-driven environments, reinforcing the relevance of nutritional context in eating disorder prevention.

Mechanistic insights strengthen the life-course framework emerging from this Research Topic. Population-based analyses from Huang et al. indicate that dietary inflammatory potential and socioeconomic disadvantage partially mediate associations between depression and gastrointestinal dysfunction. Although conducted in adults, these findings are highly relevant to adolescence, as inflammatory dietary patterns and social inequalities often originate earlier in life.

Taken together, the evidence assembled here reinforces that adolescent diet should not be conceptualized as an individual choice alone, but as the product of social inequality, family environment, and structural access to healthy foods. The bidirectional relationship between diet and mental health underscores the need for integrated strategies addressing nutrition, emotional wellbeing, and sleep simultaneously. Parents and families emerge as critical leverage points, and strengthening parental food literacy and confidence, particularly among socioeconomically disadvantaged groups, offers a promising pathway to sustainable change. Despite these advances, most evidence remains cross-sectional, highlighting the need for longitudinal and interventional studies. Adolescence is not only a period of vulnerability but a window of opportunity, and interventions addressing diet quality, emotional wellbeing, and structural inequities are likely to yield the most durable benefits into adulthood.

